# Soil and fine root-associated microbial communities are niche dependent and influenced by copper fungicide treatment during tea plant cultivation

**DOI:** 10.1093/hr/uhac285

**Published:** 2022-12-29

**Authors:** Ali Inayat Mallano, Jie Yu, Tabys Dina, Fangdong Li, Tiejun Ling, Naveed Ahmad, Jeffrey Bennetzen, Wei Tong

**Affiliations:** State Key Laboratory of Tea Plant Biology and Utilization, Anhui Agricultural University, Hefei 230036, China; Sericultural Research Institute, Anhui Academy of Agricultural Sciences, Hefei 230061, China; School of Medicine, Nazarbayev University, Nur-Sultan 020000, Kazakhstan; State Key Laboratory of Tea Plant Biology and Utilization, Anhui Agricultural University, Hefei 230036, China; School of Science, Anhui Agricultural University, Hefei 230036, China; State Key Laboratory of Tea Plant Biology and Utilization, Anhui Agricultural University, Hefei 230036, China; Institute of Crop Germplasm Resources, Shandong Academy of Agricultural Sciences, Jinan 250100, China; State Key Laboratory of Tea Plant Biology and Utilization, Anhui Agricultural University, Hefei 230036, China; Department of Genetics, University of Georgia, Athens, GA, 30602, USA; State Key Laboratory of Tea Plant Biology and Utilization, Anhui Agricultural University, Hefei 230036, China

Dear Editor,

Fungicide treatment has a profound effect on controlling plant pathogens in modern agriculture, however, it also carries the risk of undesirable outcomes. For decades, scientists have been concerned about the harmful impacts of heavy metals like copper (Cu) on crop performance and soil microorganisms. Use of various copper fungicides, like Bordeaux mixture, have been a component of conventional agricultural practices to control fungal and bacterial pathogens, especially in vineyards, tea gardens, or fruit tree orchards [9, 10]. This treatment increases the accumulation of high levels of Cu in surface soils, and despite the critical role of Cu as an essential trace element in wide biological and metabolic processes, it becomes toxic to plants when applied at high levels [4]. The regular application of copper fungicides has also been linked to affecting microbial communities at the levels of diversity [8], population structure [2], abundance, and growth [1, 3]. Understanding the undesired effects of fungicides on microorganisms’ beneficial activities is therefore important for evaluating the hazards associated with the fungicide used in agriculture. Yet, the effects of copper fungicide on full microbial communities remains relatively understudied, especially in tea plants. Thus, we herein explored the influence of Bordeaux mixture under different management regimes (raking or without raking leaf litter) on microbial communities of root, bulk soil, and rhizosphere compartments of tea plants planted in a ten-year-old tea garden. We provided insights into the ecological consequences of tea management practices that might help to identify specific fungicide treatment regimens, environmental characteristics, and microbial community members to minimize the negative environmental outcomes and optimize the positive anti-pathogen aspects of fungicide treatment.

**Figure 1:**
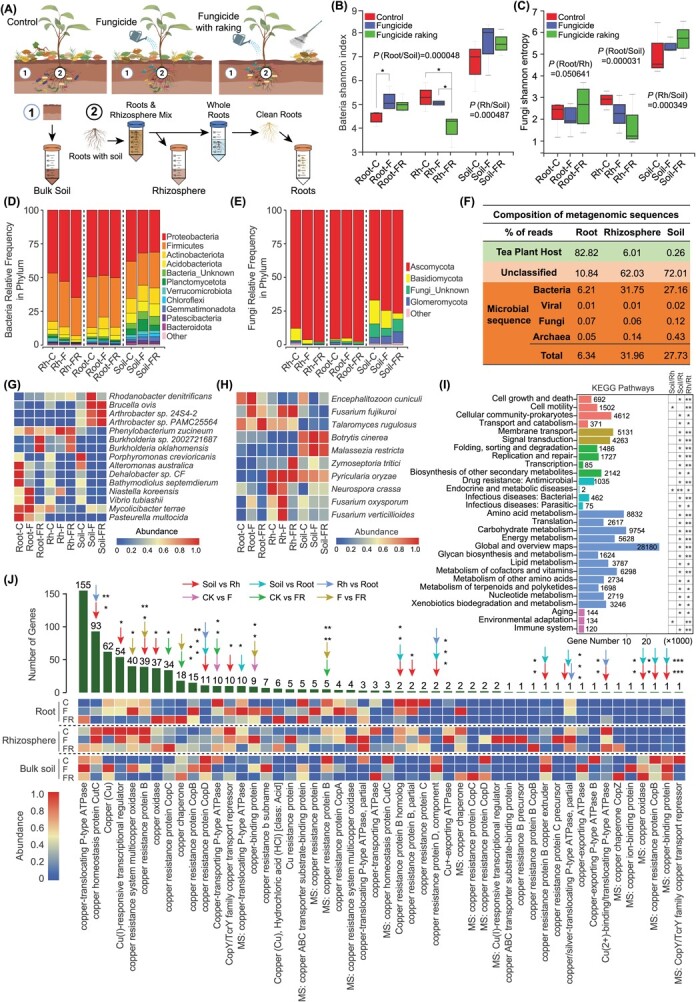
Microbial community, composition and diversity of rhizosphere, root and bulk soil compartments under fungicide treatment. **A** A graphical scheme of treatments and sample collection of tea plant microbiome in current study. Microbiomes of three compartments of tea plant including bulk soil, fine roots and rhizosphere under different conditions (control, fungicide treatment, and fungicide after raking leaf litter) were prepared and collected. **B**, **C** Alpha diversity assessment of tea plant roots, rhizosphere, and bulk soil microbiome under copper fungicide treatments. Boxplots showing the Shannon diversity for bacteria and fungi grouped by compartment. Kruskal–Wallis test was applied for the significance tests between different comparisons. ^*^P-value <0.05. **D**, **E** Bacterial and fungal community composition in the three tea plant habitats (roots, bulk soil, and rhizosphere) under fungicide treatment at phylum level. **F** Percentage of microbial sequences (bacteria, viral and fungi), plant host sequences and unclassified sequences in tea plant using reads based metagenomic sequencing. **G**, **H** Heatmaps showing the distribution of bacterial and fungal species obtained from three plant compartments root, rhizosphere, and bulk soil under fungicide treatments. The color code is representing the relative abundance ranging from high abundance (red) to low abundance (blue) grouped by treatment. **I** Functional enrichment of KEGG annotation of the genes identified in the metagenomic analysis and the significance tests between different comparisons among compartments and fungicide treatments. ^*^P-value <0.05; ^**^P-value <0.01. **J** Fifty copper-resistant gene categories identified against BacMet database across the three compartments under fungicide treatments in tea plant. The topside panel illustrates the number of genes harbored by the categories. Arrows of different colors and asterisks indicate the differential abundance copper categories among different comparisons between compartments and fungicide treatments. The lower panel heatmap indicates the overall gene abundances within each category related to copper resistance. ^*^P-value <0.05; ^**^P-value <0.01; ^***^P-value <0.001. C: control; F: fungicide treatment; FR: fungicide with raking treatment.

We assessed fine root-associated microbial communities in three-year-old field-grown tea plants treated with fungicide Bordeaux mixture (final concentration of 0.6% copper and 0.3% lime powder, with a 1:0.5:100 per liter water ratio) before and after raking leaf litter. Compartments of bulk soil, rhizosphere and fine root samples were taken after 10 days of fungicide treatment (Fig. 1A). We detected the physiochemical composition of bulk soil and found that only nitrogen, zinc, and copper, as expected, displayed a significant difference after treatment. Amplicon sequencing of bacteria and fungi were then conducted by amplifying the V3-V4 of 16S and ITS2 gene regions. After data trimming and clustering, a total of 3 886 877 and 2 197 386 reads for 16S and ITS were generated, further assigned to 13 615 bacterial and 1723 fungal RSVs (ribosomal sequence variants). Alpha diversity of different compartments under treatments varied significantly in bacteria and fungi, revealing a gradient of diversity from bulk soil to rhizosphere and then to roots (Fig. 1B and C). Fungicide treatments generally increased the bacteria and fungi alpha diversity in roots and bulk soil, but critically decreased the diversity in the rhizosphere samples (Fig. 1B and C**)**. This indicates that fungicide may affect the rhizosphere most in tea plants by directly decreasing their microbial diversity. Interestingly, the lowest alpha diversity was found in fungicide after raking treatment samples for the rhizosphere compartment of both bacteria and fungi. We then investigated the microbiome composition and abundance of different compartments samples. A total of 28 bacterial phyla and seven fungal phyla were identified. The bacteria were predominately colonized by Proteobacteria (45.90%), Firmicutes (31.21%), Actinobacteriota (7.60%), Acidobacteriota (4.80%), and unknown bacteria (2.47%), accounting for >90% of the total detected sequences (Fig. 1D). We found that Actinobacteriota and Acidobacteriota show increased abundances under fungicide treatment in root samples, but decreased patterns in the other compartments. Planctomycetota and Gemmatimonadota in soil and Proteobacteria in rhizosphere showed higher abundances under fungicide treatments, respectively. Ascomycota was the most dominant phylum across all the samples and contributed to an average abundance of 87.57% of the total fungal diversity (Fig. 1E). Fungicide treatment decreased the abundance of Ascomycota generally in all the compartments. Abundance of Basidiomycota was clearly reduced under fungicide treatment in rhizosphere and soil samples, but not in the root compartment. Glomeromycota showed low abundance in rhizosphere and root samples with a decreasing trend under fungicide, but higher abundance in soil compartment with higher amounts after fungicide treatment. This revealed that the microbial responses of tea plants after fungicide treatment in bulk soil are different from that in the root and rhizosphere.

Reads-based metagenomics of bulk soil, rhizosphere, and root compartments were also performed to validate the composition and abundances. Approximately 9.8 Gb metagenomic sequences per sample were generated. Among all the reads, an average 6.34% in root, 31.96% in rhizosphere, and 27.73% in soil were classified as microbial sequences, in which a high proportion of the microbial sequences (root: 6.21%; rhizosphere: 31.75%; soil: 27.16% of all the reads) was assigned to bacteria (Fig. 1F). This gave us a general landscape and composition of the microbial communities in tea plant roots, rhizosphere, and bulk soil. Similar to the result in amplicon sequencing, Proteobacteria (62.28%), Actinobacteria (29.39%), Firmicutes (3.33%), Acidobacteria (1.92%), and Bacteroidetes (1.45%) in bacteria and Ascomycota (92.17%), Basidiomycota (7.67%) in fungi were detected as the most abundant phylum across the samples. Species, such as *Rhodanobacter denitrificans*, *Brucella ovis*, and *Arthrobacter* sp. had clearly higher abundances in bulk soil under fungicide and fungicide with raking treatments, while low abundances were observed in rhizosphere and root compartments (Fig. 1G). *Burkholderia*, which is necessary for protein repair and turnover under copper stress and possess antagonistic properties against fungal pathogens, were enriched in rhizosphere and root under fungicide raking treatment but not in bulk soil [5, 7]. Simultaneously, fungicide boosted the abundance of *Niastella koreensis* and *Vibrio tubiashii* in the root and rhizosphere compartments, while *Alteromonas australica, Dehalobacter sp. CF* and *Bathymodiolus septemdierum*were more abundant in control samples (Fig. 1G). Fungicide treatment increased the fungal abundance of *Botrytis cinerea*, a well-known tea plant pathogen that causes Gray-mold disease [6], along with *Malassezia restricta*, only in bulk soil but not in the roots and rhizosphere (Fig. 1H). Meanwhile, *Encephalitozoon cuniculi* and *Fusarium fujikuroi* in roots, as well as *Fusarium verticillioides* and *Fusarium oxysporum* in rhizosphere were enriched and showed increased abundance under fungicide treatments compared to control (Fig. 1H).

To further investigate the functional responses of microbiota in bulk soil, rhizosphere, and root of tea plant under cupper fungicide treatment, we conducted functional assessment of the metagenomic genes in the microbial population. Functional annotations of the predicted genes using Kyoto Encyclopedia of Genes and Genomes (KEGG) identified 29 pathways. Among them 3, 27, and 29 pathways were found to be differentially enriched between soil and rhizosphere, soil and root, rhizosphere and root (*P* < 0.05); however, we didn’t find significant differences in pathways among the fungicide and raking treatments (Fig. 1I). A collection of 25 917 microbial genes associated with 922 biocide and metal-resistance categories against the BacMet database were characterized, of which 50 categories were related to copper (Fig. 1J). Among them, Copper-translocating P-type ATPase (155 genes), Copper (Cu, 62 genes), and copper homeostasis protein CutC (93 genes) were the most abundant categories (Fig. 1J). Comparative abundance analysis revealed 22 categories that were differentially presented among the fungicide treatments and different compartments (Fig. 1J). We found more differentially expressed gene categories between compartments than under the fungicide treatment, suggesting the diverse responses of tea plant root compartments against the fungicide treatment (Fig. 1J).

Herein, we provided taxonomic evidence of copper responses in tea plant under natural habitats of soil, rhizosphere, and fine root gradient. We revealed that copper fungicide treatments not only increased the abundance of bacteria, including *Rhodanobacter denitrificans*, *B. ovis*, *Arthrobacter* and phytopathogens fungi, including *Pyricularia oryzae*, *Botrytis cinerea*, *Fusarium* species, but also could suppress the abundances of fungal taxa, such as beneficial fungi *Talaromyces rugulosus*. This suggested that copper fungicide treatment induces a much more complex shift in soil-associated microbiomes than expected from a simple anti-fungal model. Together with further investigations on the response mechanisms of tea plant against fungicides, these reported findings will serve to improve crop management strategies and decrease negative environmental outcomes.

## Acknowledgments

This work was supported by the National Natural Science Foundation of China (No. 32002086), the Natural Science Research Project of University in Anhui Province (No. 202244), the Top Talent Team Project of Anhui Agriculture University (No. 03082021), and Key Program in the Joint Funds of National Natural Science Foundation of China (No U19A2034).

## Author contributions

J.B., W.T., and A.I.M. designed and supervised the study; W.T. and A.I.M. collected the samples and did the formal analysis; A.I.M., J.Y., and W.T. wrote the manuscript; F.L., T.D., J.Y., and N.A. helped to do the analysing; A.I.M., J.Y., J.B., T.L., and W.T. revised the manuscript.

## Data availability

Raw reads of the amplicon and metagenomic sequences reported in this study have been deposited into the National Center for Biotechnology Information BioProject database under accession number of PRJNA703764.

## Conflict of interest

The authors declare that they have no conflict of interest.

## References

[1] Dell'Amico E , Mazzocchi M, Cavalca L et al. Assessment of bacterial community structure in a long-term copper-polluted ex-vineyard soil. *Microbiol Res* 2008;163:671–83. 17207985 10.1016/j.micres.2006.09.003

[2] Fernández-Calviño D , Martín A, Arias-Estévez M et al. Microbial community structure of vineyard soils with different ph and copper content. *Appl Soil Ecol* 2010;46:276–82.

[3] Gobbi A , Kyrkou I, Filippi E et al. Seasonal epiphytic microbial dynamics on grapevine leaves under biocontrol and copper fungicide treatments. *Sci Rep* 2020;10:681. 31959791 10.1038/s41598-019-56741-z PMC6971271

[4] Griffiths BS , Philippot L. Insights into the resistance and resilience of the soil microbial community. *FEMS Microbiol Rev* 2013;37:112–29. 22568555 10.1111/j.1574-6976.2012.00343.x

[5] Higgins S , Gualdi S, Pinto-Carbo M et al. Copper resistance genes of burkholderia cenocepacia h111 identified by transposon sequencing. *Environ Microbiol Rep* 2020;12:241–9. 32090500 10.1111/1758-2229.12828

[6] Karakaya A , Bayraktar H. Botrytis disease of tea in Turkey. *J Phytopathol* 2010;158:705–7.

[7] Kong P , Richardson P, Hong C. Burkholderia sp. Ssg is a broad-spectrum antagonist against plant diseases caused by diverse pathogens. *Biol Control* 2020;151:104380.

[8] Nunes I , Jacquiod S, Brejnrod A et al. Coping with copper: legacy effect of copper on potential activity of soil bacteria following a century of exposure. *FEMS Microbiol Ecol* 2016;92:fiw175. 27543319 10.1093/femsec/fiw175

[9] Seenivasan S , Muraleedharan N. Cumulative effect of foliar application of copper oxychloride on pb content in black tea. *Journal of Tea Science Research* 2015;5:1–4.

[10] Wightwick AM , Walters RD, Allinson G et al. Environmental risks of fungicides used in horticultural production systems. In: Odile Carisse (Eds.), Fungicides, Chapter 14: 272-303 (InTechOpen, 2010).

